# Asymmetry of Radial and Symmetry of Tangential Neuronal Migration Pathways in Developing Human Fetal Brains

**DOI:** 10.3389/fnana.2016.00002

**Published:** 2016-01-25

**Authors:** Yuta Miyazaki, Jae W. Song, Emi Takahashi

**Affiliations:** ^1^Department of Medicine, Chiba University School of MedicineChiba, Japan; ^2^ Department of Radiology and Biomedical Imaging, Yale University School of MedicineNew Haven, CT, USA; ^3^Division of Newborn Medicine, Department of Medicine, Boston Children’s Hospital, Harvard Medical SchoolBoston, MA, USA; ^4^Athinoula A. Martinos Center for Biomedical Imaging, Massachusetts General Hospital, Harvard Medical SchoolCharlestown, MA, USA; ^5^Fetal-Neonatal Neuroimaging and Developmental Science Center, Boston Children’s Hospital, Harvard Medical SchoolBoston, MA, USA

**Keywords:** development, radial migration, ganglionic eminence, human, diffusion imaging, tractography

## Abstract

The radial and tangential neural migration pathways are two major neuronal migration streams in humans that are critical during corticogenesis. Corticogenesis is a complex process of neuronal proliferation that is followed by neuronal migration and the formation of axonal connections. Existing histological assessments of these two neuronal migration pathways have limitations inherent to microscopic studies and are confined to small anatomic regions of interest (ROIs). Thus, little evidence is available about their three-dimensional (3-D) fiber pathways and development throughout the entire brain. In this study, we imaged and analyzed radial and tangential migration pathways in the whole human brain using high-angular resolution diffusion MR imaging (HARDI) tractography. We imaged ten fixed, postmortem fetal (17 gestational weeks (GW), 18 GW, 19 GW, three 20 GW, three 21 GW and 22 GW) and eight *in vivo* newborn (two 30 GW, 34 GW, 35 GW and four 40 GW) brains with no neurological/pathological conditions. We statistically compared the volume of the left and right radial and tangential migration pathways, and the volume of the radial migration pathways of the anterior and posterior regions of the brain. In specimens 22 GW or younger, the volume of radial migration pathways of the left hemisphere was significantly larger than that of the right hemisphere. The volume of posterior radial migration pathways was also larger when compared to the anterior pathways in specimens 22 GW or younger. In contrast, no significant differences were observed in the radial migration pathways of brains older than 22 GW. Moreover, our study did not identify any significant differences in volumetric laterality in the tangential migration pathways. These results suggest that these two neuronal migration pathways develop and regress differently, and radial neuronal migration varies regionally based on hemispheric and anterior-posterior laterality, potentially explaining regional differences in the amount of excitatory neurons that migrate along the radial scaffold.

## Introduction

Corticogenesis is a complex process that occurs during a critical period of brain development. This process begins with subcortical neuroproliferation, followed by neuronal migration and the formation and refinement of axonal connections (Caviness et al., 1996). Neuronal subtypes of different origins migrate differentially along transient pathways to ultimately establish connections that result in a highly conserved, six-layered neocortical structure. In developing human brains, the radial and tangential migration pathways are two primary sources of neural progenitor cells.

Radial glia are recognized as a primary source of progenitor cells and also serve as a scaffold for migrating neurons in the developing brain. During corticogenesis, a complex process of both symmetric and asymmetric divisions of radial glial cells from the ventricular zone (VZ) has been described, with symmetric divisions attributed to increasing the progenitor pool and asymmetric divisions resulting in neuronal generation (Cai et al., 2002). Neuron subtypes that originate from the radial migration pathway include excitatory glutamatergic projection neurons and GABAergic neurons (Haubensak et al., 2004), which migrate along radial glial fascicles toward the cortical plate (CP; Rakic, 1972, 1988; Bystron et al., 2008; Petanjek et al., 2009a,b). One histologic study showed vimentin immunoreactive radial glia in the developing visual cortex in the occipital lobe of the human brain at specific pre- (14 gestational weeks (GW), 19 GW, 36 GW) and post-term (79 GW) ages, describing the fate and destination of radial glia towards this region during development (Honig et al., 1996). Other studies investigated radial pathways with immunohistochemistry at 10–19 GW (Bayatti et al., 2008) and with lipophilic tracing methods at 14–15 GW (Hansen et al., 2010). However, detailed information regarding radial glial pathways remains unknown as these studies lacked specimens from a critical period of brain development (19–36 GW).

Another major neuronal migratory pathway is the tangential migration pathway, which runs tangential to the CP and from which inhibitory GABAergic interneuron progenitors arise (Wonders and Anderson, 2006). The tangential migration pathway originates from the ganglionic eminence (GE), and although the VZ/subventricular zone (SVZ) are also suggested to be important sources of GABAergic interneurons (Radonjić et al., 2014; Al-Jaberi et al., 2015; Arshad et al., 2015), the GE has been suggested to be a major pool of GABAergic interneurons (Hansen et al., 2013; Ma et al., 2013; Keverne, 2014). Both pathways have been extensively studied in rodents (e.g., Price and Thurlow, 1988; Walsh and Cepko, 1988; Anderson et al., 1997), primates (Rakic, 1972; Radonjić et al., 2014) and to a lesser degree, humans (e.g., Rakić and Sidman, 1969; Letinic and Rakic, 2001; Al-Jaberi et al., 2015). Taken together with the literature on rodents and primates, recent studies on human fetal brains showed that certain aspects of neuronal migration are unique to human corticogenesis, emphasizing the need to examine trajectories of neuronal migration pathways directly in the developing human brain (Letinic and Rakic, 2001; Al-Jaberi et al., 2015). However, due to limitations inherent to microscopic studies, only small regions of neuronal migratory streams have been histologically investigated, and a systematic understanding of the neuronal migratory streams during the human fetal stage is still elusive.

Initial efforts to assess migratory streams include whole brain analysis by magnetic resonance imaging (MRI; Zhang et al., 2013), and analyses of hemispheric asymmetries (Snyder et al., 1995; Kovalev et al., 2003). Several groups have imaged diffusion coherence of migration pathways by conventional diffusion tensor imaging (DTI) tractography (McKinstry et al., 2002; Huang et al., 2009), but many questions still remain regarding the time-course of the development and regression of the migratory streams as well as the need for comprehensive analyses utilizing whole brain specimens and wider age ranges. Moreover, conventional DTI methods are challenged by the reconstruction of crossing neural fibers in the brain (Tuch et al., 2003).

High-angular resolution diffusion imaging (HARDI) tractography is a technique that enables reconstruction of complicated crossing neural fibers in the brain (Tuch et al., 2003), even in immature brains (Takahashi et al., 2011, 2012, 2013, 2014; Song et al., 2014). Given the technical advantage of HARDI tractography, the present study aims to analyze radial and tangential migration pathways in developing human brains using this technique to understand patterns of neuronal migration during a critical period of brain development.

Disorganized neuronal migration from the radial and tangential pathways has been implicated in many neurological and psychiatric disorders (Marín and Rubenstein, 2001; Gressens, 2006; Volpe, 2009) such as intractable epilepsy (Cusmai et al., 2014), developmental dyslexia (Adler et al., 2013; Platt et al., 2013), autism (Wegiel et al., 2010), and schizophrenia (Benes and Berretta, 2001; McIntosh et al., 2008; Hori et al., 2014; Muraki and Tanigaki, 2015). Thus, a better understanding of this complex developmental process is critical to gain further insight into such disease processes.

## Materials and Methods

### Fetal Brain Specimens

Ten fetal brain specimens were imaged for this study. IRB committees at Partners and Boston Children’s Hospital (BCH) approved the use of postmortem specimens for MRI studies. The brains were acquired after obtaining informed consent by the parents. Table 1 reports the details of GW, the sources of specimens, and whether the brain was imaged *ex-* or *in vivo*. Seven *postmortem* brain specimens were provided by the Department of Pathology, Brigham and Women’s Hospital (BWH; Boston, MA, USA), and three *postmortem* brain specimens were obtained from the Allen Institute Brain Bank (AIBB; Seattle, WA, USA). These brains were grossly normal, and standard autopsy examination of all brains undergoing *postmortem* HARDI revealed minimal or no pathologic abnormalities at the macroscopic level. Any cases with suspected abnormality, malformations and disruption were excluded from this study. *Ex vivo* fetal and postnatal brains and living postnatal infant brains were included in the study, which allowed us to reveal brain structure in unforeseen detail. Although *ex vivo* imaging is superior, we have previously shown that *ex*- and *in vivo* imaging produces comparable tracking results in brains at the fetal, neonatal, and toddler ages (Xu et al., 2014).

**Table 1:** **Characteristics of brain specimens**.

Subject	Source	Age	Brain image	Age group
1	BWH	17 GW	*Ex vivo*	Early
2	BWH	18 GW	*Ex vivo*	Early
3	AIBB	19 GW	*Ex vivo*	Early
4	BWH	20 GW	*Ex vivo*	Early
5	BWH	20 GW	*Ex vivo*	Early
6	BWH	20 GW	*Ex vivo*	Early
7	BWH	21 GW	*Ex vivo*	Early
8	AIBB	21 GW	*Ex vivo*	Early
9	BWH	21 GW	*Ex vivo*	Early
10	AIBB	22 GW	*Ex vivo*	Early
11	BCH	30 GW	*In vivo*	Late
12	BCH	30 GW	*In vivo*	Late
13	BCH	34 GW	*In vivo*	Late
14	BCH	35 GW	*In vivo*	Late
15	BCH	40 GW	*In vivo*	Late
16	BCH	40 GW	*In vivo*	Late
17	BCH	40 GW	*In vivo*	Late
18	BCH	40 GW	*In vivo*	Late

*BWH, Brigham and Women’s Hospital; AIBB, Allen Institute Brain Bank; BCH, Boston Children’s Hospital; GW, Gestational week*.

### *In Vivo* Subjects

Eight living participants underwent clinically-indicated brain MRI studies that were interpreted to show no abnormalities. The IRB at BCH approved the retrospective use of *in vivo* imaging data. Indications for imaging included concern for hypoxic ischemic injury, apnea and transient choreiform movements after an upper respiratory tract infection. None had clinical concerns for a congenital malformation or genetic disorder. All MR image acquisitions were performed under protocols approved by each hospital’s institutional review board for human research.

### Tissue Preparation for HARDI

At the time of autopsy, all brains were immersion fixed. The brains from BWH were stored in 4% paraformaldehyde, and the brains from AIBB were stored in 4% periodate-lysine-paraformaldehyde (PLP). During MR image acquisition, BWH brains were placed in Fomblin solution (Ausimont, Thorofare, NJ, USA; Takahashi et al., 2012), and AIBB brains were placed in 4% PLP. While these different solutions tend to change the background contrast (i.e., a dark background outside of the brain is often visualized using Fomblin, and a bright background using PLP), these solutions do not specifically change diffusion properties (e.g., FA and ADC) within the brain parenchyma (Kolasinski et al., 2013; Song et al., 2014).

### Diffusion MRI Procedures

Six postmortem brain specimens from BWH were imaged with a 4.7T Bruker Biospec MR system, and three specimens from the AIBB were imaged with a 3T Siemens MR system at the A. A. Martinos Center, Massachusetts General Hospital, Boston, MA, USA. The 3T system was used to accommodate the AIBB brains that were *in cranio* and did not fit in the 4.7T bore. To improve the imaging quality and obtain the best signal-to-noise and high spatial resolution, we used custom-made radio-frequency (RF) coils with one channel on the 4.7T and 3T systems (Takahashi et al., 2012; Xu et al., 2014). We used multiple scanner systems to utilize the best-fit RF coils to ensure optimal imaging with good signal to noise.

For the BWH brains, a three-dimensional (3-D) diffusion-weighted spin-echo echo-planar imaging (SE-EPI) sequence was used with a repetition time/echo time (TR/TE) of 1000/40 ms, with an imaging matrix of 112 × 112 × 112 pixels. Sixty diffusion-weighted measurements (with the strength of the diffusion weighting, *b* = 8000 s/mm^2^) and one non-diffusion-weighted measurement (no diffusion weighting or *b* = 0 s/mm^2^) were acquired with *δ* = 12.0 ms and *Δ* = 24.2 ms. The spatial resolution was 440 × 500 × 500 μm. For the brains from the AIBB, diffusion-weighted data were acquired over two averages using a steady-state free-precession sequence with TR/TE = 24.82/18.76 ms, *α* = 60°, and the spatial resolution was 400 × 400 × 400 μm. Diffusion weighting was isotropically distributed along 44 directions (*b* = 730 s/mm^2^) with 4 *b* = 0 images. We determined the highest spatial resolution for each brain specimen with an acceptable signal-to-noise ratio of more than 130 for tractography.

The brains of living participants were imaged on a 3T Siemens MR system, BCH, Boston, MA, USA. The diffusion pulse sequence used was a diffusion-weighted SE-EPI sequence, TR/TE 8320/88 ms, with an imaging matrix of 128 × 128 × 64 pixels. The spatial resolution was 2 × 2 × 2 mm. Thirty diffusion-weighted measurements (*b* = 1000 s/mm^2^) and five non-diffusion-weighted measurements (*b* = 0 s/mm^2^) were acquired with *δ* = 40 ms and *Δ* = 68 ms.

### Reconstruction and Identification of Tractography Pathways

We used Diffusion Toolkit and TrackVis^1^ to reconstruct and visualize neural migration pathways. A streamline algorithm for diffusion tractography was used (Mori et al., 1999), described in previous publications (Schmahmann et al., 2007; D’Arceuil et al., 2008; Takahashi et al., 2010, 2011, 2012). The term “streamline” refers to connecting tractography pathways using a local maximum or maxima. This method is true for both DTI and HARDI. The streamline technique is limited in its ability to resolve crossing pathways when used with the traditional DTI technique because one simply connects the direction of the principal eigenvector on a tensor to produce the DTI tractography pathways. This feature is a recognized limitation of DTI (Mori et al., 1999). Hence, in the current study, we used HARDI, which can theoretically detect multiple local maxima on an orientation distribution function (ODF). Using each local maxima on an ODF, we applied the streamline algorithm to initiate and continue tractography (Tuch et al., 2003), thus enabling us to identify crossing pathways within a voxel.

Trajectories were propagated by consistently pursuing the orientation vector of least curvature. Tracking was terminated when the angle between two consecutive orientation vectors was greater than the given threshold (40°) or when the fibers extended outside of the brain surface using a brain mask. The brain mask volumes were used to terminate tractography structures instead of the FA threshold (Schmahmann et al., 2007; Wedeen et al., 2008; Takahashi et al., 2010, 2012; Vishwas et al., 2010), because progressive myelination and crossing fibers in the developing brain can result in low FA values and the use of an FA threshold may potentially incorrectly terminate tractography tracking in regions with low FA values.

### Regions of Interest (ROIs)

**Figure 1:**
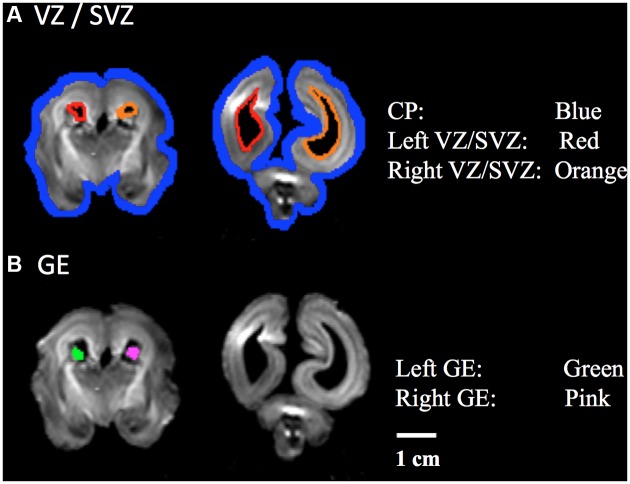
**Regions of Interest (ROIs) used to identify radial and tangential tractograpy pathways. (A)** ROIs placed in cortical plate (CP, blue) and ventricular/subventricular zone (VZ/SVZ; left: red, right: orange), **(B)** ROIs placed in a middle part of ganglionic eminence (GE; left: green, right: pink).

Using previously reported methodology (Takahashi et al., 2012; Kolasinski et al., 2013), a regions of interest (ROIs) approach was used to identify radial and tangential tractogrpahy pathways. ROIs for the CP, VZ/SVZ, and GE were identified (Figures 1A,B). We used ROIs of the region of VZ/SVZ and CP for identification of the radial migration pathway (Figure 1A). Tangential pathways were identified using ROIs of the GE (Figure 1B). Previous studies also have shown the GE structure by manually identifying its location (Huang et al., 2009; Kostovic and Vasung, 2009; Takahashi et al., 2012; Song et al., 2014).

### Statistical Analyses

Volume measurements are automatically derived by TrackVis for each migration pathway. The volume of pathways was calculated by counting the number of voxels that touched or passed through the detected pathways, and was compared at different developmental time points.

We defined left and right radial pathways separately between the VZ/SVZ and CP in each hemisphere. We also defined the anterior and posterior radial pathways as the front or posterior half of the radial pathway. Due to challenges in defining the “middle” coronal plane in early fetal stages from only anatomic information without gyral or other mature subcortical structures, we instead measured the length between the anterior and posterior edges of the brain and defined a middle coronal plane to separate the anterior and posterior radial pathways. Left/right hemispheric asymmetry was tested for both the radial and tangential migration pathways, and anterior-posterior differences were tested for the radial pathway.

We statistically analyzed asymmetry with a laterality index (LI), calculated by the following formula:

$${\text{LI}}_{\text{LR}}\text{=}\left(\text{L}-\text{R}\right)/\{\text{0}.\text{5}\times \left(\text{L+R}\right)\}\text{for comparison of Left and Right hemispheres}$$

$${\text{LI}}_{\text{AP}}\text{=}\left(\text{A}-\text{P}\right)/\{\text{0}.\text{5}\times \left(\text{A+P}\right)\}\text{for comparison of Anterior and Posterior regions}$$

LIs range from −2 to 2, with positive and negative LI values corresponding to left- and rightward, and anterior and posterior asymmetries, respectively (Caviness et al., 1996). The asymmetries of the radial and tangential migration pathways using LIs were statistically analyzed with SPSS version 19.0.0 (IBM SPSS, Chicago, IL, USA). Statistical significance was set at *P* < 0.01.

## Results

### Qualitative Description of the Development and Regression Time-Lines of Radial and Tangential Migration Pathways

**Figure 2:**
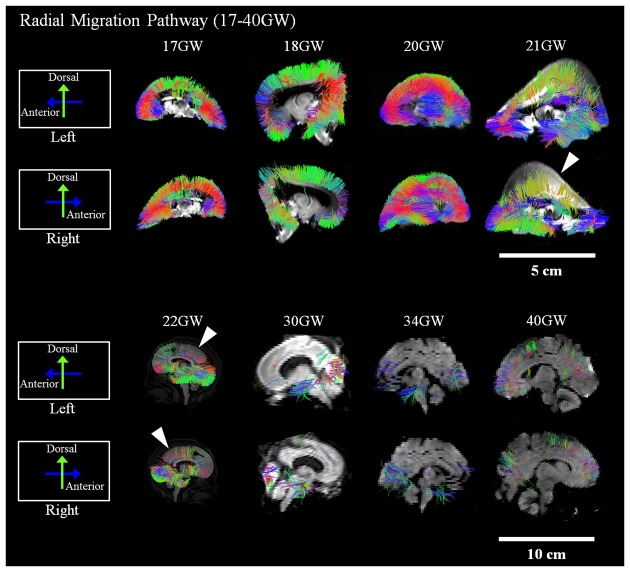
**Radial pathways.** Tractography at representative ages of the radial pathways are shown in sagittal views. White arrowheads show brain regions where the radial pathways became progressively sparse. Scale bar = 5 cm for 17–21 GW, and 10 cm for 22–40 GW.

**Figure 3:**
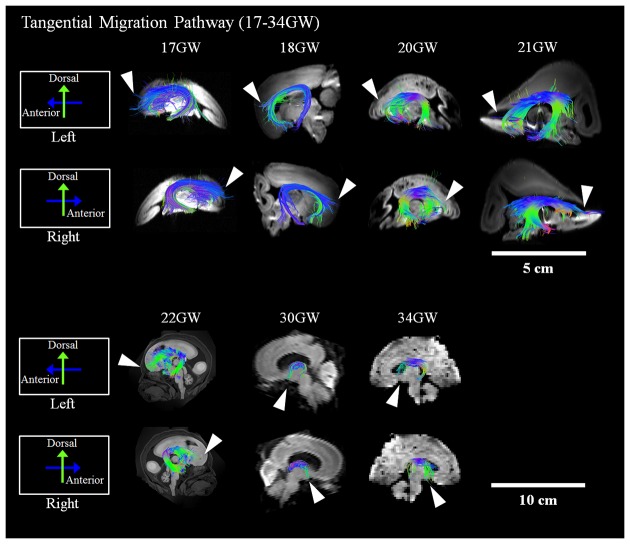
**Tangential migration pathways.** Tractography at representative ages of the tangential migration pathways are shown in sagittal views. White arrowheads show examples of the tangential migration tract branching into the cerebral walls. Scale bar = 5 cm for 17–21 GW, and 10 cm for 22–40 GW.

Development and regression of the radial and tangential migration pathways are shown in Figures 2, 3. Radial and tangential migration pathways were both identified in the early age group (17–22 GW), while only radial pathways were identified in the late age group (30–40 GW). The tangential migration pathways in the late age group (30 and 34 GW) became sparse, and after 34 GW they were not identified by tractography.

#### Radial Migration Pathways

Figure 2 shows reconstructed radial tractography pathways (perpendicular to the cortical mantle) running through the cerebral wall in the sagittal views (Figure 2). Radial pathways gradually decreased density by 21 GW (Figure 2, white arrowheads). Residual fiber pathways were still observed at 40 GW in a few brains suggestive of individual variability. Gradual disappearance of the radial pathways was not evenly observed in the whole brain. The disappearance of radial pathways by tractography started in the dorsal parietal and superior frontal regions at 21 GW and progressed in an anterior, inferior direction towards the inferior frontal lobe after 21 GW (Figure 2, white arrowheads). At 30 GW, a large segment of the radial pathways persisted in the occipital lobe. However, by 40 GW, the density of the radial pathways appeared substantially decreased, globally.

#### Tangential Migration Pathways

Reconstructed GE tractography pathways are shown in Figure 3. Pathways likely corresponding to the GE migration stream were very thick at 17 GW and revealed some radial pathways emanating from the stream at the most distal anterior and posterior regions as it penetrated the cortical mantle (Figure 3, white arrowheads). These radial pathways were contiguous with tangentially oriented pathways, as previously described by Kolasinski et al. (2013). Until 22 GW, tangential migration pathways appeared tightly packed with gradual thinning in the sagittal planes (Figure 3). After 30 GW, the tangential migration pathways became sparser, and after 34 GW, the tangential migration pathways were not identifiable by tractography (Figure 3, white arrowheads).

### Quantitative Results

#### Hemispheric Asymmetry

**Figure 4:**
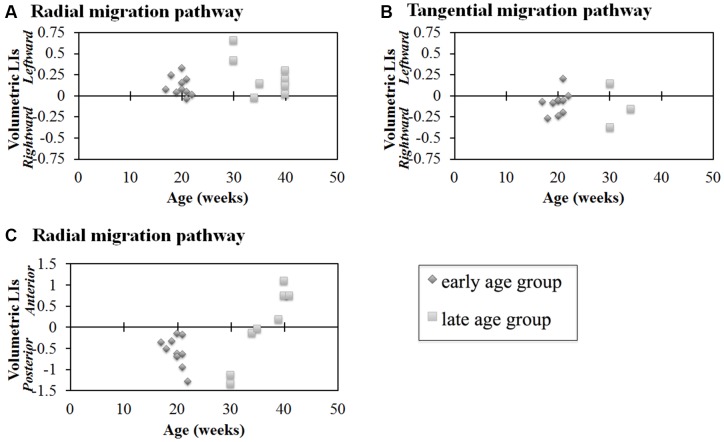
**Laterality indices (LIs) for hemispheric asymmetry (A,B) and anterior-posterior asymmetry (C) of radial pathways (A,C) and tangential pathways (B).** Radial pathways in the early age group revealed significant hemispheric asymmetry (*p* = 0.0045) **(A)**. In contrast, significant hemispheric asymmetry was not observed in the tangential pathways (*p* > 0.01) **(B)**. In the early age group, the posterior region was significantly larger than the anterior region (*p* < 0.0001, [1.75 × 10^−5^]) **(C)**. In contrast, no significant difference was observed in the late age group **(C)**. Diamond shape: early age group (17–22 GW); square: late age group (30–40 GW).

Next, we studied the volumetric laterality indices (LIs) for the radial and tangential migration pathways in both the early and late ages (Figures 4A,B). Significant leftward asymmetry in the radial pathways in the early age group was observed (*p* = 0.0045). In contrast, asymmetry in the late age group was not observed (*p* > 0.01). When combining all ages, significant leftward asymmetry of the radial pathways emerged (*p* = 0.0019). By contrast, statistically significant hemispheric asymmetry was not observed in any age groups for the GE pathway (*p* > 0.01).

#### Anterior-Posterior Differences

Results of comparisons between the volume of the anterior and posterior regions of the identified radial pathways are shown in Figure 4C. In the early age group, the posterior region was significantly larger than the anterior region (*p* < 0.01). In contrast, no significant difference was observed in the late age group of the radial pathway (*p* > 0.01). Combining all ages, no significant volumetric difference was observed between the anterior and posterior regions of the radial pathways (*p* = 0.55).

## Discussion

We investigated two major neural migration pathways, the radial and tangential migration pathways, in human fetal and newborn brains (17–40 GW) using HARDI tractography. Due to limitations of microscopy, previous studies have performed only limited regional analyses of these migration pathways in developing human brains. Our new findings are: (1) significant left-right hemispheric asymmetry and anterior-posterior asymmetry of radial migration pathways between 17 GW and 22 GW by analyzing whole human fetal brain with MR imaging; (2) there were no significant asymmetry of tangential migration pathways between 17 GW and 22 GW, and no significant asymmetry of radial migration pathways between 30 GW and 40 GW. Our previous study (Kolasinski et al., 2013) reported on the radial and tangential migration pathways, but the study just used three specimens between 19 GW and 21 GW and qualitatively described trajectories of pathways without investigating asymmetry. These results suggest that these two neuronal migration pathways develop and regress differently. This study showed, for the first time, hemispheric and anterior-posterior asymmetry of neuronal migration pathways in human fetal brains.

### Radial Migration Pathway—Development and Regression Time Line

Many lines of evidence suggest that the majority of radial pathways contribute excitatory glutamatergic projection neurons (Rakic, 1972, 1988; Bystron et al., 2008; Petanjek et al., 2009a,b). Therefore, the volume of the radial pathway may, in part, reflect the relative degree of density of this subtype of glutamatergic neurons originating from this pathway. Our study revealed that the disappearance of radial pathways began at 21 GW. This thinning of radial tractography pathways was most conspicuous in the dorsal parietal and frontal regions and progressed to the inferior frontal lobe around 22 GW. This observation suggests a determined spatiotemporal direction of migration of excitatory neurons that arise from the radial pathway.

### Radial Migration Pathway—Hemispheric Asymmetry

While the radial tractography pathways showed structural asymmetries, the tangential migration pathways revealed no significant hemispheric asymmetry in volume in this study. These results suggest that, at least in the pathways that supply inhibitory interneurons to the cortex, there is no demonstrable laterality by tractography.

The volume of the radial pathway in the left hemisphere was significantly larger than that of the right hemisphere in the early age group (17–22 GW) in our study. In contrast, no significant difference between the left- and right hemispheres was observed in the late age group (30–40 GW). Previous studies reported that the left hemisphere is volumetrically larger than the right hemisphere in right-handed adults (Büchel et al., 2004), and it has been suggested that the leftward asymmetry in Broca’s area contributes to human language acquisition (Schenker et al., 2010). Such normal hemispheric asymmetry in adults is often not observed in neurological and psychiatric disorders (e.g., Mackay et al., 2003). However, to date, very few studies have explored the developmental origins of this asymmetry. Although handedness was not confirmed, a larger left temporal area was already observed in the fetal stage by 30 GW (Chi et al., 1977). Even earlier, leftward asymmetry of gyral folding in specific temporal areas started to be observed from 23 GW (Habas et al., 2012). By term (or term-equivalent ages), leftward asymmetries were also detected in some tractography pathways likely corresponding to the superior longitudinal fasciculus, thalamocortical pathways, and the corticospinal tract (Liu et al., 2010). Given the link between neuronal migration and gyral formation in cobblestone lissencephaly (Dobyns et al., 1996), classical lissencephaly (Glesson, 2000; Lammens, 2000; Leventer et al., 2000), and polymicrogyria (Chang et al., 2004; Guerrini and Filippi, 2005; Jansen and Andermann, 2005), one can speculate that the leftward volumetric asymmetry of radial neuronal migration pathways may contribute to hemispheric asymmetry of the number of neurons in the cortex, followed by hemispheric asymmetry of axonal pathways from the neurons. In fact, the earliest gestational age where Habas et al. (2012) found hemispheric asymmetry of gyral structures (23 GW) coincides with the early age group of the current study (17–22 GW) when we observed leftward asymmetry. Future studies are necessary to directly link the transient leftward asymmetry of the radial pathway to the hemispheric asymmetry in mature brains.

### Radial Migration Pathway—Anterior-Posterior Asymmetry

In the current study, the volume of the radial pathway in the posterior region was significantly larger compared to the anterior region when examining ages up to 22 GW in our study. This quantitative analysis was in agreement with the visual tractography of the radial pathways, which revealed a gradual thinning of this pathway in the parieto-occipital regions at 21 GW. This thinning of pathways appeared to progress towards the frontal region by 22 GW. Previous studies reported a higher neural density in the posterior than in the anterior brain regions in primates (Cahalane et al., 2012; Charvet et al., 2015), presumably in adult mature brains, although the studies did not specify the ages of the brains. Our result suggests that a higher density of the radial tractography pathways in the posterior region contributes to the anterior-posterior gradient in neuron density.

While the regression of the radial pathways in the dorsal parietal and frontal regions continued through 21 GW, the regression of the radial pathways in the inferior frontal regions seemed to begin after 22 GW. Although radial pathways in the occipitoparietal regions also decreased in these ages, the late-onset regression in the inferior frontal regions seemed greater than in the occipitoparietal regions, which likely contributed to the relative increase in volume of the posterior radial pathways compared to those in the anterior region. At 30 GW, almost no anterior radial pathways were visible, while there were some residual radial pathways in the posterior regions. By 34 GW and 40 GW, radial pathways almost all disappeared throughout the brain. One reason why no significant difference between the anterior and posterior regions was observed in the late age group may be due to the uneven number of specimens in each age group within the late age group. For instance, in the late age group, only 30 GW (*n* = 2 of 8) brains revealed radial pathways remaining in the posterior regions, yet 40 GW brains (*n* = 4 of 8), which revealed sparse to no pathways in the posterior regions, comprised half of the specimens in this late age group. For future studies, increasing the number of brains and evening out the distribution of ages, specifically of specimens in the 23–29 GW, would be important to gain a more comprehensive understanding of the regression of radial migration pathways.

### Tangential Migration Pathway

The tangential migration pathways were observed in the early age group (17–22 GW) and at the beginning of the late age group (30–34 GW), but were not visible at 40 GW. This observation is consistent with the literature describing the gradual disappearance of the GE by 34 GW (Rakić and Sidman, 1969). Many lines of evidence in primate and human studies report that a substantial percentage of cortical GABAergic neurons arise from the GE (Letinic et al., 2002; Rakic and Zecevic, 2003; Yu and Zecevic, 2011) with the peak of GABAergic neurogenesis occurring at the end of the second trimester of gestation (Petanjek et al., 2008; 15–24 GW). Similarly, in our study, the tangential migration pathways were not distinctly identifiable by tractography in brains developed past the second trimester. This finding is consistent with the literature reporting this pathway as a transient anatomic structure. However, during the early ages of development when the GE was identifiable by tractography, no significant asymmetry was observed.

While Arshad et al. (2015) claimed that interneuron generation in the GE extends beyond 35 GW (not PCW, however), we only showed tangential tractography pathways until 34 GW in the current study. A limitation of our study is a sampling gap between 34–40 GW. Thus it is possible that tractography may have been detectable past 34 GW, if samples were available. Another potential reason for these controversial results is the age grouping in the study by Arshad et al. (2015). They grouped 29–35 GW and 35–40 GW together. The only figure that they showed for the results from the 35–40 GW group Figure 6, Arshad et al. (2015) is not really convincing regarding the existence of interneuron generation at this age range. Other figures showing results only until 35 GW showed some evidence for interneuron generation, but because 35 GW was grouped with 29–34 GW, it is possible that a younger population primarily contributed to their results.

The coherent diffusivity within the GE visualized by tractography during the early fetal ages is likely attributable to the tangential migratory routes on scaffolds of corticofugal fibers. This directionality of migration has been shown in investigational studies using tracer-labeling (Denaxa et al., 2001) and fate mapping studies (Wichterle et al., 2001). These studies have shown that during the period of corticogenesis, migrating neurons follow multiple tangential routes to their destination in the developing cortex. More specifically, cells from the lateral GE migrate ventrally and anteriorly to give rise to neurons in the striatum. Cells from the medial GE have been shown to migrate dorsally and posteriorly (Wichterle et al., 2001). The temporospatial migratory pattern likely contributes to the diffusivity seen within the GE in this study. Although many studies confirming this tangential migratory pattern have been performed in mice, similar findings have also been described in humans (Hansen et al., 2013; Ma et al., 2013) and further support our tractography findings in human fetal brains.

## Conclusion

In this study, we examined whether volumetric asymmetries by tractography were present in two critical neuronal migratory pathways, the radial and tangential migration pathways, from which progenitor neurons have been reported to migrate in the process of corticogenesis in developing human fetal brains. Several conclusions may be drawn from this study. First, our results confirm a postero-dorsal to antero-ventral direction of migration of the radial pathway based on the gradual disappearance of tractography pathways. By tractography, the volume of radial pathways was greater in the posterior region than in the anterior brain region at early stages of development (22 GW and younger). Second, volumetric asymmetry for only the radial pathways was statistically significant in the early age group (22 GW and younger). Third, no volumetric hemispheric asymmetry of the tangential pathway emerged, contrasting with the radial pathway. Taken together, these findings suggest hemispheric volumetric asymmetry related to neural progenitors from the radial glial pathway is established early in development, which contrasts with the GE. Describing the anatomic development and regression of these neuronal migratory pathways is critical as many neurologic and psychological disorders are described to have underlying disorganized corticogenesis. Future investigations aim to include a more comprehensive range of developmental stages to enable a more thorough description of the developmental changes, as well as comparisons of normal and diseased brains to understand what may contribute to the underlying problems in the formation of brain connectivity.

## Author Contributions

ET performed the experiments. YM, JWS, and ET analyzed the data and wrote the paper.

## Conflict of Interest Statement

The authors declare that the research was conducted in the absence of any commercial or financial relationships that could be construed as a potential conflict of interest.

## Footnotes

^1^http://trackvis.org
